# Study on the Multiple Efficacies of Vitamin C Serum in Anti‐Glycation, Anti‐Carbonylation, Antioxidation, and Anti‐Inflammation of Human Skin Based on In Vivo Tests

**DOI:** 10.1111/jocd.70888

**Published:** 2026-05-05

**Authors:** Yusha Zi, Jianwei Liu, Qi Liu, Yao Pan, Xiuyu Jiang

**Affiliations:** ^1^ Amway (Shanghai) Technology Development Co., Ltd. Shanghai China; ^2^ Beijing EWISH Testing Technology Co., Ltd. Beijing China; ^3^ Department of Cosmetics, School of Light Industry Science and Engineering Beijing Technology and Business University Beijing China

## Abstract

**Background:**

Skin glycation, oxidation, carbonylation, and excessive inflammation are well‐recognized factors contributing to skin aging and pigmentation. Previous in vitro studies have confirmed vitamin C's antioxidant, anti‐glycation, and anti‐inflammatory properties, but its in vivo effects remain to be further verified.

**Aims:**

To investigate the in vivo effects of topical vitamin C serum on skin anti‐glycation, anti‐carbonylation, antioxidation, and anti‐inflammation, and to provide evidence for its anti‐aging and skin‐brightening applications.

**Patients/Methods:**

A randomized, double‐blind, controlled trial was conducted, enrolling 66 healthy Chinese females, with 31 in the blank group and 35 in the topical group. Participants applied 10% vitamin C serum for 12 weeks. Skin glycation, carbonylated protein content, interleukin‐1α (IL‐1α) levels, free radical scavenging capacity, and skin color parameters were evaluated before and after treatment.

**Results:**

After 12 weeks, the topical group showed significant improvements: AGEs reduced by 17.65%, carbonylation fluorescence intensity decreased by 49.22%, IL‐1α content dropped by 58.73%, and ABTS free radical scavenging rate increased by 12.14%. Skin yellowness (b* value) and redness (a* value) decreased by 6.13% and 16.46%, respectively (all *p* < 0.001).

**Conclusions:**

Topical 10% vitamin C serum can effectively mitigate skin glycation, carbonylation, and inflammation, enhance skin antioxidant capacity, and improve skin color, supporting its clinical value in anti‐aging and skin‐brightening.

## 1 Introduction

Skin aging and pigmentation represent two of the most common dermatological concerns among adult populations, with profound impacts on individuals' aesthetic satisfaction and psychological well‐being. Beyond the natural chronological aging process, environmental stressors (e.g., ultraviolet radiation and pollution) and physiological imbalances further accelerate these phenotypes, driving the need to identify and target their underlying molecular mechanisms. In recent decades, accumulating evidence has established that four interrelated biological processes, including skin glycation, oxidation, carbonylation, and excessive inflammatory factor release, act as core contributors to the pathogenesis of skin aging and pigmentation [1, 2].

Skin glycation refers to the non‐enzymatic reaction between reducing sugars and proteins, such as collagen and elastin, which leads to the formation of advanced glycation end products (AGEs) [3]. Over time, these AGEs accumulate in the dermis, promoting cross‐linking of structural proteins, impairing mechanical elasticity, and inducing oxidative stress. These changes ultimately result in visible signs of aging, including skin sagging, fine lines, and dullness. Concurrently, oxidative stress arises from an imbalance between reactive oxygen species (ROS) production and antioxidant defenses. This imbalance causes direct damage to cellular membranes, DNA, and lipids within skin cells, accelerating cellular senescence and stimulating melanocytes to produce excess melanin, thereby contributing to pigmentation disorders. Skin carbonylation, a consequence of persistent oxidative stress, involves the modification of proteins by reactive carbonyl species (RCS) [4]. Carbonylated proteins lose their biological function and accumulate in the stratum corneum, leading to increased skin roughness, dryness, and a yellow complexion [5]. Furthermore, the excessive release of pro‐inflammatory cytokines, including interleukin‐1α (IL‐1α), disrupts skin barrier function, amplifies oxidative damage, and sustains a state of chronic low‐grade inflammation [6]. This inflammatory environment further aggravates aging‐related skin conditions [7]. Importantly, a complex biochemical crosstalk exists among glycation, oxidation, and inflammation in the skin. Advanced glycation end products (AGEs) can bind to their specific receptor (RAGE) on epidermal cells, activating downstream signaling pathways that directly induce the expression and release of mature IL‐1α from keratinocytes [8, 9]. Concurrently, oxidative stress and resulting protein carbonylation further stimulate IL‐1α secretion, which, in a feedback loop, amplifies the generation of reactive oxygen species (ROS) [10]. This chronic, low‐grade inflammatory state, driven synergistically by glycation and oxidation—termed ‘inflammaging’—is a core mechanism accelerating skin structural degradation [11]. Therefore, quantifying IL‐1α in the stratum corneum serves not only as a direct measure of the epidermal micro‐inflammatory state but also as a highly sensitive, downstream biomarker reflecting the cumulative damage from both glycation and carbonylation.

Against this backdrop, VC (vitamin C) has emerged as a promising candidate for mitigating skin aging and pigmentation, owing to its well‐documented biological activities. In vitro studies have demonstrated that VC can scavenge ROS directly to restore redox balance, inhibit the formation of AGEs by competing with sugars for protein binding sites [12], and suppress the expression of pro‐inflammatory cytokines in cultured keratinocytes [13]. Moreover, VC has been shown to reduce protein carbonylation by neutralizing RCS, thereby preserving the structural and functional integrity of skin proteins [14]. Despite these compelling in vitro findings, the translational value of such results remains limited. Most existing studies have been conducted in isolated cell cultures or cell‐free systems, which fail to replicate key aspects of the complex physiological microenvironment in human skin. As a result, the actual efficacy of VC in modulating glycation, oxidation, carbonylation, and inflammation in human skin remains insufficiently validated, creating a critical gap between laboratory research and clinical application.

To address this gap, the present study was designed to systematically investigate the effects of VC on key pathological processes: skin glycation, oxidation, and carbonylation through a rigorous in vivo human trial. By leveraging clinically relevant assessment methods such as the AGE Reader for glycation and tape stripping for stratum corneum sampling, this research aims to validate the multifunctional efficacy of VC under physiological conditions.

## 2 Methods

### 2.1 Product Information

Directions for use: The topical formulation contains 10% VC as the sole active ingredient. The vehicle consists of water, propylene glycol, pentylene glycol, and glycerin. In detail, the 10% VC formulation was prepared as a powder vehicle dual chamber system to maximize stability. VC powder was stored separately from the liquid vehicle and mixed by participants immediately before each use. Apply morning and evening. The powder and vehicle were separately packaged in opaque, light‐proof containers to prevent oxidation and light degradation. Participants were clearly instructed to store both components in a cool, dark place, avoid direct sunlight and high temperatures, and mix fresh before application to ensure optimal activity throughout the 12‐week study period. After cleansing and toning your face each day, apply 4–5 drops to the entire face, avoiding direct contact with the eyes.

The study recruited healthy Chinese female volunteers aged between 18 and 60. All participants were office workers and had skin concerns such as dryness, dullness, hyperpigmentation, lack of radiance, and sagging. Initially, 35 subjects were assigned to the control group, of whom 31 completed the trial. In the topical treatment group, 35 subjects were recruited, and all 35 completed the study, bringing the total number of participants who completed the study to 66. Subjects in the topical treatment group received topical treatment with 10% VC for 12 consecutive weeks. The study period was from 15 April 2024 to 5 August 2024. The protocol for each study was approved by the Shanghai Clinical Research Ethics Committee. All volunteers signed informed consent forms. Participation in the study was entirely voluntary, and subjects could withdraw from the study at any time. The ethical review approval number for this project is SECCR/2024‐46‐01.

#### 2.1.1 Assessments

#### 2.1.2 Anti‐Glycation

Advanced glycation end products (AGEs) in the skin were measured using the AGE Reader (Diagnoptics, Netherlands) at a precisely defined anatomical site: the intersection of a horizontal line extending from the tip of the nose to the subject's cheek and a vertical line descending from the centre of the pupil. Three consecutive measurements were taken at this specific location, with the mean value used for analysis. The measured parameter represents the ratio of the emitted fluorescence (420–600 nm) to the reflected excitation light (300–420 nm). A higher value indicates a greater level of skin glycation, whereas a lower value corresponds to reduced glycation. The results are expressed in arbitrary units (AU).

#### 2.1.3 Anti‐Carbonylation

Participants thoroughly rinsed their cheeks with tap water and patted the skin surface dry. A commercial adhesive tape (D‐SQUAME D100) was firmly applied to the center of the cheek for 5 s using a pressure applicator (D‐SQUAME Pressure Instrument, CuDerm#D500), and then peeled from the skin. To eliminate the interference of surface sebum, residual cosmetics, and environmental contaminants, this first tape strip was routinely discarded. A subsequent tape strip was then collected from the exact same site using the identical standardized procedure. The retained tape was stained with fluorescein‐5‐thiosemicarbazide (Thermo Fisher Scientific) to detect carbonylated proteins. Stained images were observed under a stereo fluorescence microscope (OLYMPUS IX73). To ensure quantitative accuracy and eliminate potential photographic artifacts, all images were acquired using strictly standardized imaging parameters across all subjects and time points. Specifically, a standard fluorescence filter set for fluorescein was utilized (excitation wavelength: 485 nm; emission wavelength: 530 nm) [15]. The exposure time was locked at a constant 200 ms, and the camera gain was maintained at 1.0 ×. All auto‐exposure and auto‐enhancement functions were completely disabled. According to the established in situ visualization and quantitative method for tape‐stripped stratum corneum [15], specific regions containing valid stratum corneum (SC) cells were selected for image analysis. The average fluorescence intensity (mean pixel intensity) of these specific SC regions was then quantified, and the mean value was reported as the level of stratum corneum carbonylated protein (SCCP).

#### 2.1.4 Anti‐Inflammation

Following the acquisition of skin samples using the aforementioned tape‐stripping method, the collected tape strip was placed into a 1.5 mL tube containing RIPA lysis buffer (Beyotime Biotechnology). The sample was subjected to ultrasonication for 10 min to efficiently extract the stratum corneum proteins. After centrifugation to remove the tape and cellular debris, the supernatant was collected. The total protein concentration of the extract was determined using a BCA Protein Assay Kit prior to cytokine analysis. Subsequently, the level of Interleukin‐1 alpha (IL‐1α) was determined using a commercial enzyme‐linked immunosorbent assay kit (Interleukin‐1 alpha, Thermo Fisher Scientific), strictly in accordance with the manufacturer's instructions. The final results were expressed as IL‐1α content per unit of total protein.

#### 2.1.5 Anti‐Oxidant

Following the collection of skin samples using the aforementioned tape‐stripping method, the stratum corneum proteins were extracted using the identical RIPA lysis and ultrasonication protocol described in Section 2.1.3. Because this standardized extraction process yields a uniform sample solution correlated with the total extracted protein (quantified via BCA assay), the ABTS assay kit (Beyotime Biotechnology) was subsequently used on this extract according to the manufacturer's instructions. The measurements included the preparation of standards, preparation of the ABTS working solution, and determination of the radical scavenging rate. The ABTS free radical scavenging rate (D_VC_%) was calculated using the following equation: D_VC_% = [A_blank_—(A_sample_—A_control_)]/A_blank_*100%, where A_blank_ is the absorbance of the blank control, A_sample_ is the absorbance of the test sample, and A_control_ is the absorbance of the sample itself without the ABTS working solution to account for background interference. Because a standardized extraction process was used for all subjects, the resulting scavenging rate (%) directly reflects the standardized antioxidant capacity of the individual's sampled stratum corneum.

#### 2.1.6 Skin Color

Skin color was measured using a CM‐26d spectrophotometer (Konica Minolta, Japan) on a precisely defined anatomical site: the intersection of the horizontal line extending from the tip of the nose and the vertical line descending from the center of the pupil on the subject's cheek. Three consecutive measurements were taken at this specific site, and the average value was used for analysis. Three consecutive measurements were taken at each site, and the average value was used for analysis. The a* value represents the red‐green balance of the skin. A higher a* value indicates a more pronounced red tone in the skin. The b* value represents the yellow‐blue balance. A higher b* value indicates a more pronounced yellow tone in the skin.

### 2.2 Statistical Analysis

Graphs were generated using GraphPad, with results expressed as mean ± standard deviation. Comparisons within each group before and after treatment were performed using the paired sample *t*‐test, wheras the Wilcoxon signed‐rank test was applied for data that did not follow a normal distribution. A *p*‐value of less than 0.05 was considered statistically significant.

## 3 Results

Prior to evaluating the specific efficacy markers, the demographic characteristics of the participants were analyzed to ensure baseline comparability. Among the subjects who completed the 12‐week study, the mean age of the blank control group (*n* = 31) was 50.13 ± 6.76 years, and the mean age of the topical treatment group (*n* = 35) was 49.11 ± 7.70 years. An independent samples *t*‐test revealed no significant difference in chronological age between the two groups (*p* = 0.574). Furthermore, as shown in (Table 1), no significant differences were observed between the two groups across any of the baseline molecular or phenotypic skin parameters (*p* > 0.05). This comprehensive baseline balance validates that the subsequent improvements observed in the treatment group are attributable to the topical VC intervention, negating the need for further age‐adjusted covariate analysis.

**TABLE 1:** Summary of clinical skin parameters (mean ± SD) at baseline and after 12 weeks of treatment.

Indicator	Control group (*n* = 31)		Treatment group (*n* = 35)	
	BL	W12	BL	W12
Skin AGEs	2.19 ± 0.52	2.17 ± 0.45	2.72 ± 0.55	2.24 ± 0.50
Fluorescence intensity of carbonylated proteins	69.71 ± 2.92	69.48 ± 2.52	69.67 ± 2.27	35.38 ± 2.12
IL‐1α concentration	37.48 ± 15.60	37.03 ± 15.06	37.85 ± 0.48	15.62 ± 0.90
ABTS free radical scavenging rate	14.40 ± 2.67	13.51 ± 1.98	13.67 ± 2.84	15.79 ± 3.02
Skin b* values	15.68 ± 2.31	16.84 ± 2.08	16.47 ± 1.81	15.46 ± 1.76
Skin a* values	10.31 ± 1.79	13.66 ± 1.63	13.73 ± 1.64	11.47 ± 1.72

### 3.1 Anti‐Glycation

**FIGURE 1:**
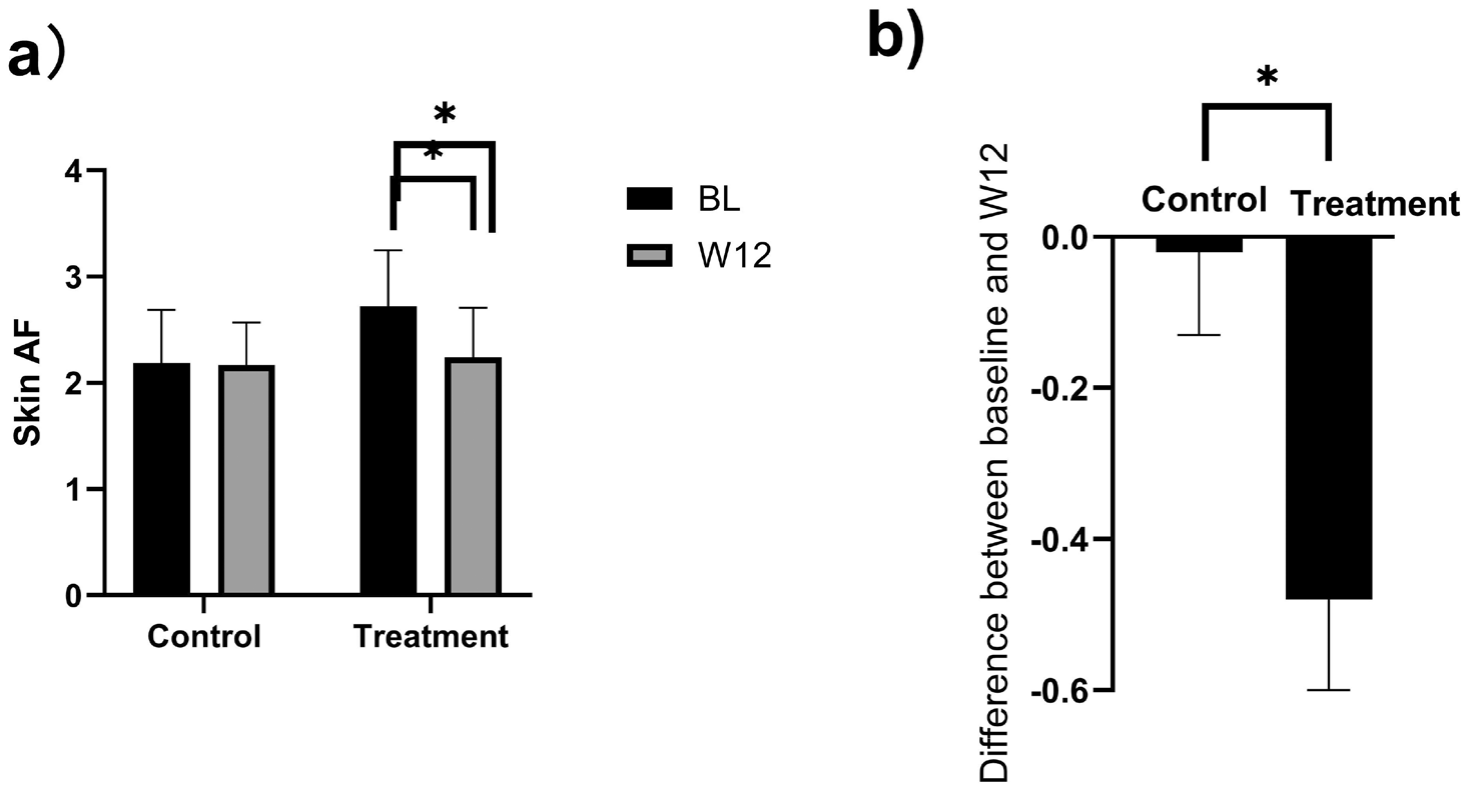
(a) Changes in skin glycation levels before and after product use; (b) degree of improvement in glycation levels following product use.

At baseline, no significant difference in glycation levels was observed between the two groups (*p* = 0.087). By week 12, the blank control group showed minimal glycation change with a−0.91% change rate relative to baseline (*p* = 0.763). In contrast, the topical VC group had a significant glycation reduction with a−17.65% change rate relative to baseline (Figure 1a; *p* < 0.001). Between‐group comparison at week 12 revealed the topical VC group had significantly lower glycation levels (Figure 1b; *p* < 0.001), confirming its anti‐glycation efficacy.

### 3.2 Anti‐Carbonylation

**FIGURE 2:**
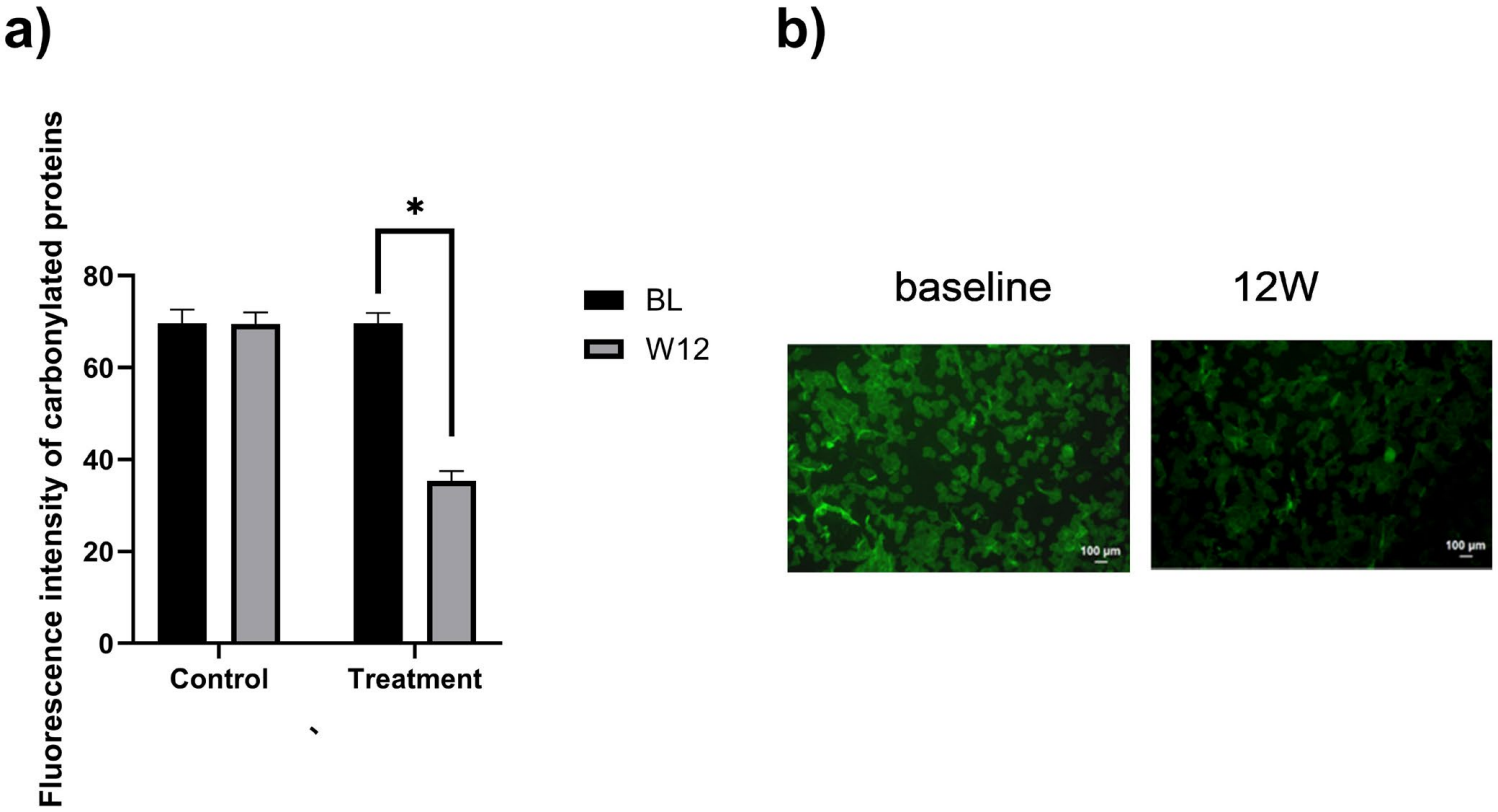
(a) Changes in skin carbonylation levels before and after product use; (b) representative fluorescence images (images obtained from Subject No. 25 in the treatment group).

At baseline, no significant difference in carbonylation levels existed between the two groups (*p* = 0.921). By week 12, the blank control group showed minimal carbonylation change with a−0.33% change rate relative to baseline (*p* = 0.815). In contrast, the topical VC group had a significant carbonylation reduction with a−49.22% change rate relative to baseline (Figure 2a; *p* < 0.001). Between‐group comparison at week 12 confirmed the topical VC group had significantly lower carbonylation levels (*p* < 0.001), verifying its anti‐carbonylation efficacy.

### 3.3 Anti‐Inflammation

**FIGURE 3:**
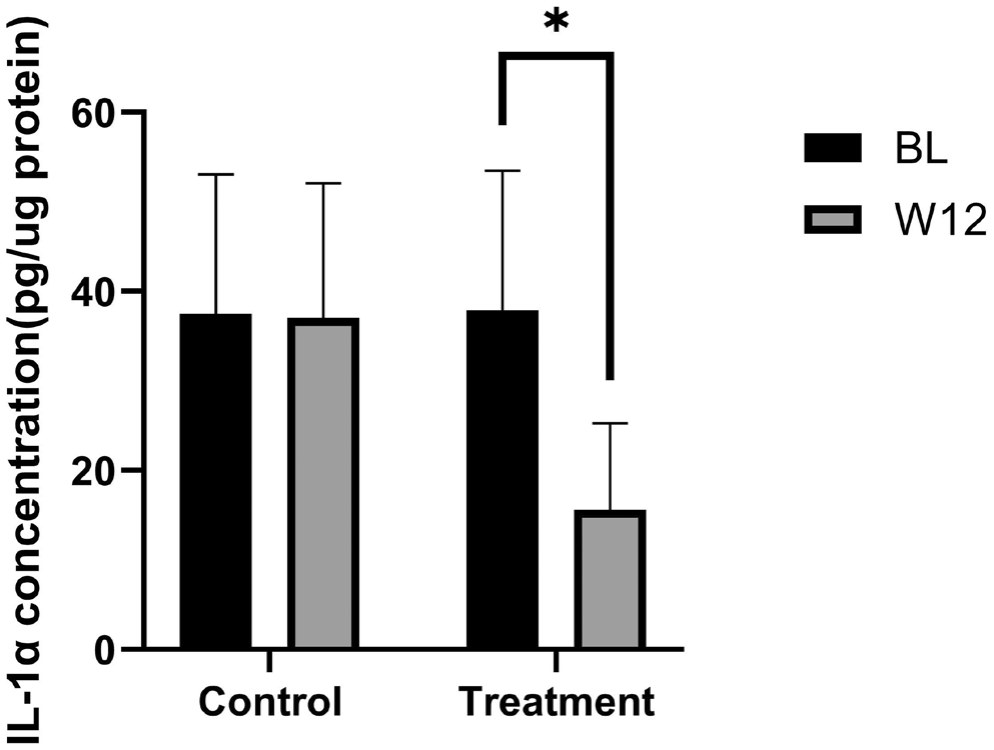
Changes in skin IL‐1α levels before and after product use.

At baseline, no significant difference in IL‐1α levels existed between the two groups (*p* = 0.896). By week 12, the blank control group showed minimal IL‐1α change with a−1.20% change rate relative to baseline (*p* = 0.783). In contrast, the topical VC group had a significant IL‐1α reduction with a−58.73% change rate relative to baseline (Figure 3; *p* < 0.001). Between‐group comparison at week 12 confirmed the topical VC group had significantly lower IL‐1α levels (*p* < 0.001), verifying its anti‐inflammatory efficacy.

### 3.4 Anti‐Oxidant

**FIGURE 4:**
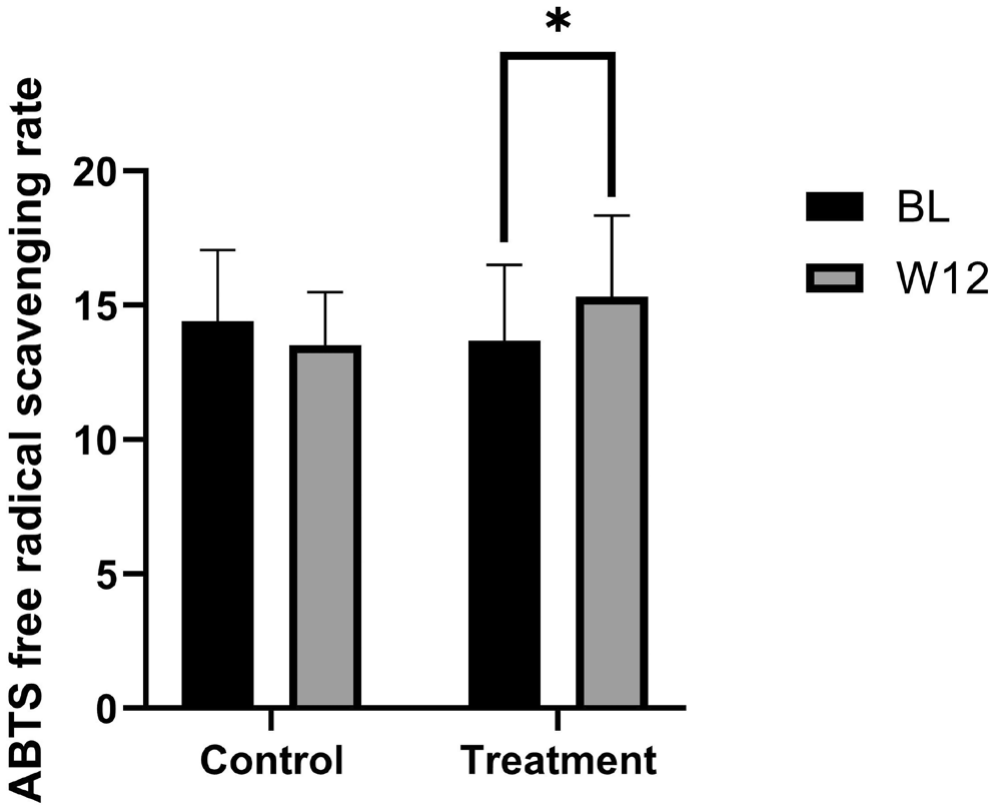
Changes in free radical scavenging rate before and after product use.

At baseline, no significant difference in scavenging rate existed between the two groups (*p* = 0.903). By week 12, the blank control group showed a slight decrease with a−6.18% change rate relative to baseline (*p* = 0.728). In contrast, the topical VC group had a significant increase with a 12.14% change rate relative to baseline (Figure 4, *p* < 0.001). Between‐group comparison at week 12 confirmed the topical VC group had a significantly higher scavenging rate (*p* < 0.001), verifying its ability to enhance skin antioxidant capacity.

### 3.5 Skin Color

**FIGURE 5:**
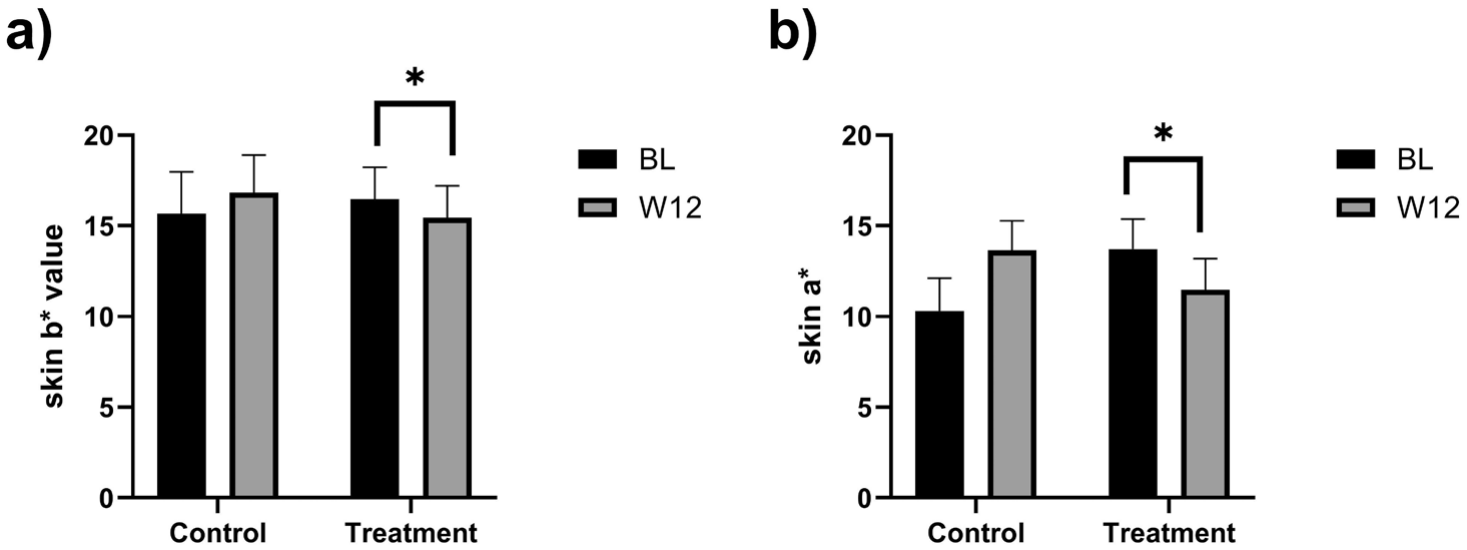
Changes in skin color before and after product use: (a) b* values; (b) a* values.

At baseline, no significant difference in b* value existed between the two groups. By week 12, the blank group showed an increase in b* value with a 7.40% change rate relative to baseline (Figure 5a; *p* < 0.001). In contrast, the topical group had a significant decrease in b* value with a−6.13% change rate relative to baseline. Between‐group comparison at week 12 confirmed the topical group had a significantly lower b* value than the blank group (*p* < 0.001), verifying the topical group's efficacy in reducing skin yellowness.

At baseline, no significant difference in a* value existed between the two groups. By week 12, the blank group showed an increase in a* value with a 32.49% change rate relative to baseline (Figure 5b; *p* < 0.001). In contrast, the topical group had a significant decrease in a* value with a−16.46% change rate relative to baseline. Between‐group comparison at week 12 confirmed the topical group had a significantly lower a* value than the blank group (*p* < 0.001), verifying the topical group's efficacy in reducing skin redness.

## 4 Discussion

Skin aging and abnormal skin color are complex processes driven by interconnected biological mechanisms, among which glycation, oxidation, carbonylation, and excessive inflammatory factors play mutually reinforcing roles. Skin oxidation, triggered by an imbalance of reactive oxygen species (ROS), not only directly damages cell membranes and DNA to accelerate senescence but also acts as an upstream driver of carbonylation [16]. This occurs because prolonged oxidative stress generates reactive carbonyl species, which modify proteins into dysfunctional carbonylated forms, thereby worsening skin roughness and sallowness. Meanwhile, oxidation amplifies inflammatory responses by promoting pro‐inflammatory cytokine (e.g., IL‐1α) release, which disrupts the skin barrier and further exacerbates oxidative damage [17]. Glycation, forming advanced glycation end products (AGEs), not only cross‐links collagen to reduce skin elasticity but also induces additional ROS production, creating a vicious cycle with oxidation, carbonylation, and inflammation. Collectively, these processes synergistically lead to visible aging signs, including sagging and wrinkles, as well as abnormal skin coloration, such as redness resulting from inflammation and yellowness caused by glycation and carbonylation, making them key targets for anti‐aging and skin‐brightening interventions.

Although in vitro studies have long highlighted VC's potential in mitigating these processes, such as by scavenging ROS, competing with sugars to inhibit AGE formation, neutralizing RCS, and suppressing inflammatory cytokine expression, these findings cannot fully translate to clinical efficacy. In vitro systems, including isolated cell cultures or cell‐free models, lack the complex physiological microenvironment of human skin, such as intercellular communication, intact barrier function, and systemic metabolic regulation, leading to inconsistencies between laboratory results and real‐world effects [18]. Addressing this gap, the present study employed an innovative tape‐stripping method to collect stratum corneum samples, combined with rigorous in vivo measurements (e.g., AGE Reader and ABTS assay). The results confirmed that topical Vc significantly reduced skin glycation, oxidative stress, carbonylated protein content, and inflammatory factor IL‐1α. It is important to note that although topical VC primarily prevents the de novo formation of AGEs rather than degrading existing cross‐links, the continuous application over 12 weeks (covering multiple stratum corneum turnover cycles) allows the highly glycated outer corneocytes to naturally desquamate. These are gradually replaced by newer cells with significantly lower glycation levels, resulting in a net reduction of measured AGEs. More importantly, improvements in molecular indices were accompanied by enhanced skin color, as evidenced by the reduction in the b* value aligning with decreased glycation and carbonylation, which are key drivers of skin yellowness [19], and the decrease in the a* value correlating with alleviated inflammation, a major cause of skin redness [20]. In this study, the skin color parameters (a* and b* values) in the blank control group exhibited slight fluctuations over the 12‐week period. This is expected as the test site (the cheek) is a UV‐exposed area subjected to daily environmental stressors and seasonal variations. The natural progression of photo‐oxidation and environmental stress typically exacerbates skin redness and yellowness. In contrast, the topical VC group demonstrated significant improvements despite the same environmental exposure, underscoring the serum's robust protective and corrective efficacy in real‐world clinical settings. Additionally, it should be noted that the baseline IL‐1α levels measured in the stratum corneum (approximately 40 pg/μg) were relatively high even in healthy subjects. This is physiologically consistent with the role of IL‐1α as a primary epidermal ‘alarmin’. Keratinocytes constitutively synthesize and store large quantities of IL‐1α within the superficial layers to serve as a rapid first‐line defense against physical or chemical barrier disruptions [21]. Furthermore, although our subjects were systemically healthy, they presented with clinical skin concerns such as dryness and dullness, indicating a sub‐clinical state of barrier stress that naturally elevates stratum corneum IL‐1α storage [22]. These findings provide direct in vivo evidence for VC's multifunctional efficacy, bridging the gap between laboratory research and clinical application.

This study has certain limitations. First, the sample was limited to healthy Chinese females aged 18–60 years, lacking representation of males, other ethnicities, or individuals with skin conditions, which may restrict the generalizability of the conclusions. Future studies should expand the participant pool to include diverse populations to validate VC's universal efficacy. Second, the present research focused on phenotypic and basic molecular indices, without exploring underlying mechanisms at the omics level. Integrating lipidomics and proteomics in future work could identify specific lipid metabolites or proteins regulated by VC, revealing more comprehensive molecular pathways and providing deeper insights into its biological actions. Thirdly, although this study provided robust evidence for VC in mitigating the biochemical drivers of skin aging (glycation, carbonylation, oxidation, and inflammation) and improving colorimetric phenotypes, it did not directly quantify macro‐structural anti‐aging indicators, such as skin elasticity or wrinkle depth. It is well established that the accumulation of AGEs cross‐links dermal collagen, leading to a loss of elasticity, whereas carbonylation impairs the functional integrity of structural proteins. Therefore, the significant reduction of these upstream biochemical markers observed in this study theoretically lays the foundation for structural rejuvenation. Future long‐term clinical trials incorporating objective topological and biomechanical measurements (e.g., 3D wrinkle imaging and cutometry) are warranted to comprehensively correlate these molecular improvements with macroscopic anti‐aging benefits. Despite these limitations, this study offers robust in vivo evidence for topical VC's role in anti‐aging and skin‐brightening, supporting its application in skincare products.

## Author Contributions

**Yusha Zi:** main author, responsible for the development of the research proposal and wrote the main manuscript text. **Qi Liu:** collected experimental data and analyzed the data. **Yao Pan:** collected experimental data and analyzed the data. **Xiuyu Jiang:** revising the manuscript. **Jianwei Liu:** the initiator and sponsor of the research. All authors have read and approved the final manuscript.

## Funding

The authors have nothing to report.

## Ethics Statement

This study was conducted in accordance with the Declaration of Helsinki, approved by the Shanghai Ethics Committee for clinical research (approval number: SECCR2024‐46‐01) and registered in the Chinese Clinical Trial Registry (registration number: ChiCTR2400085413).

## Conflicts of Interest

The authors declare no conflicts of interest.

## Data Availability

The data that support the findings of this study are available on request from the corresponding author. The data are not publicly available due to privacy or ethical restrictions.
